# Prevalence of Overweight and Obesity Among Schoolers in Selected Schools in Ranchi, Jharkhand

**DOI:** 10.7759/cureus.81937

**Published:** 2025-04-09

**Authors:** Litna George, Ragini Singh, Pratima Kumari, Prempunita Kerketta, Rajmati Kumari, Novita Kumari, Pooja Kumari

**Affiliations:** 1 College of Nursing, Rajendra Institute of Medical Sciences, Ranchi, IND

**Keywords:** jharkhand, obesity, overweight, prevalence, schoolers

## Abstract

Introduction

Worldwide obesity among children, especially among schoolers, has drastically increased over the years. The condition is the same in India, one of the largest democracies in the world. We conducted this study to understand the current state of obesity among schoolers in India and the factors that contribute to it.

Methods

This cross-sectional survey was done to determine the prevalence of obesity and investigate the connection between different underlying factors and obesity among schoolers at multiple private schools in Ranchi, Jharkhand, from January 2024 to September 2024. Height, weight, and BMI were calculated by using a calibrated weighing scale and stadiometer. A sociodemographic pro forma was used to determine the underlying factors contributing to obesity and overweight, while a standard questionnaire evaluated the related factors. Descriptive statistics were employed to assess the frequency and percentage of sociodemographic characteristics and the prevalence of obesity and overweight, whereas correlation coefficients and linear regression were used to find any relationship between sociodemographic determinants and overweight and obesity.

Results

The research revealed that the majority of the samples were predominantly female, representing 51.6%. Hinduism comprised 89.3% of the samples. Moreover, 51.6% of the samples were from the fifth grade. A notable 92.7% of the samples were from non-tribal ethnicities. The average BMI of pupils was 18, with an SD of 11.4. We found 2.8% of the total students as overweight, 59.5% as underweight, and 37.7% as normal weight in our survey. There was a strong link between BMI and variables like eating processed foods and ethnicity (processed foods: R = 0.135, R² = 0.0181, F = 5.30, p = 0.022; ethnicity: R = 0.132, R² = 0.0175, F = 5.10, p = 0.025).

Conclusions

This study revealed a gradual increase in obesity, especially in regions previously characterized by significant undernutrition. This study validates the notable disparities in obesity rates between tribal and nontribal ethnic groups and has a direct link with the consumption of processed food. The study’s findings highlight the necessity for additional research in this domain.

## Introduction

The WHO defines overweight/obesity as an abnormal or excessive buildup of fat that endangers health [1]. A BMI beyond 25 is classified as overweight, whereas a BMI surpassing 30 is categorized as obese [2]. Overweight and obesity in children aged 5-19 years are defined as overweight when the BMI for age exceeds 1 SD above the WHO growth reference median and obesity when it exceeds 2 SDs above the WHO growth reference median.

Obesity and being overweight affect individuals regardless of age and gender [1]. Studying these conditions in schoolchildren aged 6-12 years is especially important, as this stage of life involves rapid physical growth and provides a critical window for establishing healthy habits [3]. Habits formed during this period tend to persist into adulthood, emphasizing the importance of promoting healthy behaviors early to prevent long-term health consequences [4].

The global prevalence of childhood obesity has reached alarming levels, posing a significant threat to the health, well-being, and future prospects of millions of children worldwide [5]. According to the WHO, overweight among school-age children (6-12 years) is a growing concern [1]. In 2016, 17.4% of children in this age group were classified as overweight globally [1]. Regionally, the prevalence was 14.4% in South Asia and 12.4% in Southeast Asia [6]. In terms of obesity, the WHO reported that 6-8% of children aged 6-12 years were obese worldwide in 2016 [1]. South Asia and Southeast Asia reported obesity rates of 4.5% and 3.5%, respectively, for this age group during the same period [1].

In India, country-specific data indicate a concerning rise in childhood obesity [6]. Among individuals aged 5-19 years, the prevalence of obesity ranged from 3.6% to 11.7%, with projections estimating an increase of 17 million cases by 2025 [7]. Additionally, obesity in this age group rose from 2.5% in 2015-2016 to 4.2% in 2019-2020 [1]. These figures highlight the urgent need for effective interventions to combat childhood obesity and encourage healthy lifestyles among children, both in India and globally [1].

At the local level, studies conducted in Jharkhand in 2022 revealed even higher prevalence rates [7]. Researchers found a staggering 30.2% of schoolers in the Ormanjhi block, Ranchi, to be overweight or obese [7]. Similarly, in Jamshedpur, Jharkhand, 15.5% of schoolchildren were overweight, and 6.1% were obese [8]. Despite these studies indicating a significant prevalence of obesity and overweight among schoolchildren, it is remarkable to observe a state like Jharkhand, previously characterized by a high incidence of undernutrition in children, transform into a hub of childhood obesity. This study aims to determine the prevalence of overweight and obesity, along with their related determinants, among schoolers in Jharkhand in order to assess if the incidence of these conditions is indeed rising.

## Materials and methods

Study design and participants

This cross-sectional analytical study was conducted at various private schools under the Ranchi municipal corporation, under the jurisdiction of Jharkhand State in India. Assuming that obesity would be more common among the urban population and children from upper-middle-class families, researchers opted for only private schools.

The collection of data took place between July 1, 2024 and September 30, 2024. After obtaining the complete list of schools under Ranchi Municipal Corporation, the researchers conveniently selected two schools from the list. Permission was obtained from the principals of the concerned schools, and consent and assent were taken from parents and students after giving them proper details about the study. The study included participants aged between 6 and 13 years whose parents were willing to give permission. We excluded participants who had major ailments or were not present during the data collection period. The sample size was calculated by using the OpenEpi online application [9], and finally, 289 samples were included by keeping a 10% dropout rate and 2% precision.

The outcomes of this study were being overweight or obese, and the exposures were the presence of underlying diseases, the samples’ food preferences, sleeping patterns (including when they went to bed and when they woke up), screen time, any family history of obesity, the games they played, and how often they ate junk food.

Covariates in the present study were age in years, religion, class in study, ethnicity, education status of the father and mother, occupation status of the father and mother, and socioeconomic status of the family. Data were collected using a pretested, structured questionnaire that contained sociodemographic parameters, factors influencing obesity and overweight, a calibrated, standardized digital weighing machine, and a stadiometer.

The questionnaire was validated by distributing it to seven experts and making necessary changes based on the experts’ opinions. The reliability of the questionnaire was done using Cronbach’s alpha, finding it to be 0.8.

Researchers made three visits to the schools and selected participants studying from first standard to sixth standard by collecting the samples from each school, 124 and 165, respectively. We gave the questionnaire to each student, but some had trouble with the baseline information about their parents’ education, occupation, and family income. To help, we let them take the questionnaire home and asked them to get assistance from their parents. Weight was measured by having the participants stand barefoot and upright on the weighing machine, and height was measured by having the participants stand barefoot and horizontally on a stadiometer. Finally, we calculated the BMI using a WHO growth reference specific to age and gender [10].

This study received ethical clearance from the Institutional Ethics Committee at Rajendra Institute of Medical Sciences, Ranchi, India (ECR/769/INST/JH/2015/RR-21), and to ensure the quality of the study, the researchers followed the STrengthening the Reporting of OBservational studies in Epidemiology (STROBE) reporting guideline [11].

Statistical analysis

We used Jamovi’s version 2.3.28 to analyze the study. We used descriptive statistics like frequency and percentage to evaluate the sociodemographic variables and determine the frequency of fast-food consumption among school students. To find out the relationship between various sociodemographic variables, factors affecting overweight and obesity, and BMI, we conducted a subgroup analysis. We further established the relationship between sociodemographic factors and obesity using linear regression. Quantitative variables such as age in years were categorized as 7-10 years and 11-13 years, as the latter category falls under adolescent groups [12]. To avoid bias, one researcher who was not involved in the study selection process has done the statistical analysis.

## Results

Sociodemographic characteristics

The analysis indicated that a total of 146 (50.8%) samples belonged to the age group of 7-10 years old and were primarily female, accounting for a total of 149 (51.6%). Hinduism accounted for 258 (89.3%) of the samples. A total of 149 samples (51.6%) were in the fifth grade. Significantly, 268 (92.7%) of the samples exhibited non-tribal characteristics. A significant portion of the children’s parents possessed diplomas and higher degrees, with 270 (93.4%) of fathers and 230 (79.9%) of mothers, respectively. Interestingly, the majority of the 141 samples (48.8%) belonged to the upper middle class (Table 1).

**Table 1:** Sample characteristics (N = 289)

Variable	n (%)
Age (years)	
7-10	146 (50.8)
11-13	143 (49.4)
Gender	
Male	140 (48.4)
Female	149 (51.6)
Religion	
Hindu	258 (89.3)
Muslim	26 (9.0)
Christian	2 (0.7)
Others	3 (1.0)
Class in study	
2	5 (1.7)
3	7 (2.4)
4	51 (17.6)
5	149 (51.6)
6	77 (26.6)
Ethnicity	
Tribal	21 (7.3)
Non-tribal	268 (92.7)
Education status of the father	
Diploma and above	270 (93.4)
Primary school to high school	19 (6.5)
Education status of the mother	
Diploma and above	230 (79.9)
Primary school to high school	59 (20.5)
Socioeconomic status of the family	
Upper class	24 (8.3)
Upper middle class	141 (48.8)
Lower middle	108 (37.4)
Upper lower	16 (5.5)

Prevalence of obesity and overweight among school-age children

**Figure 1:**
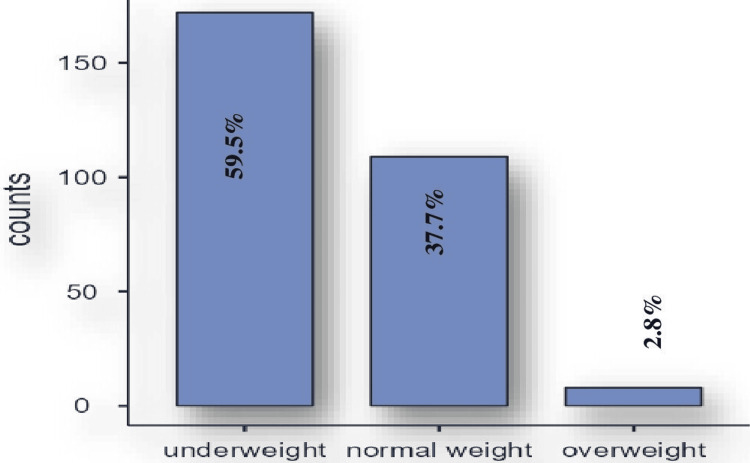
Prevalence of obesity and overweight among school-age children

The mean ± SD of BMI among schoolers in our study was 18 ± 11.4. In our study, we found only eight (2.8%) of the total 289 students as overweight, 172 (59.5%) as underweight, and 109 (37.7%) as normal weight (Figure 1).

Factors associated with obesity in school-age children

Among all samples, 12 samples (4.2%) exhibited underlying disease conditions. A total of 225 (77.8%) of respondents preferred to have non-vegetarian food options. Moreover, 173 samples (59.9%) exhibited a sleep duration of fewer than eight hours. Notably, 272 (94.1%) of individuals reported having screen time exceeding one hour per day. Only 28 samples (9.7%) reported a family history of obesity. A total of 228 samples (79%) engaged in indoor games for 30 minutes to two hours per day, while 325 samples (77.2%) participated in outdoor games for the same duration (Table 2).

**Table 2:** Factors associated with overweight and obesity (N = 289)

Variable	n (%)
Presence of underlying disease condition	
Yes	12 (4.2)
No	277 (95.8)
Food preference	
Vegetarian	64 (22.1)
Non-vegetarian	225 (77.8)
Sleeping pattern	
Less than eight hours	173 (59.9)
More than eight hours	116 (40.1)
Screen time	
Less than one hour	17 (5.9)
One to two hours	158 (54.7)
More than two hours	114 (39.4)
Family history of obesity	
Yes	28 (9.7)
No	261 (90.3)
Frequency of playing indoor games	
No	52 (18)
Thirty minutes to two hours	228 (79)
Three to five hours	9 (3)
Frequency of playing outdoor games	
No	61 (21.1)
Thirty minutes to two hours	320 (77.2)
Three to five hours	5 (1.7)

Consumption of processed food and beverages

A total of 20 samples (6.9%) consumed processed food daily, while 91 samples (31.5%) reported weekly consumption, and 136 samples (47.1%) consumed it monthly. In contrast, 19 samples (6.6%) consumed beverages daily, 86 samples (29.8%) consumed them weekly, and 109 samples (37.7%) consumed them monthly (Table 3).

**Table 3:** Consumption of processed food and beverages (N = 289)

Variable	n (%)
Consumption of processed food	
Daily	20 (6.9)
Weekly	91 (31.5)
Monthly	136 (47.1)
Never	42 (14.5)
Consumption of beverages	
Daily	19 (6.6)
Weekly	86 (29.8)
Monthly	109 (37.7)
Never	75 (26)

Correlation between BMI and various sociodemographic factors

Correlation analysis was conducted to examine the relationship between BMI and various sociodemographic factors. The results indicated a significant relationship between BMI and ethnicity (r = 0.132, p = 0.025) (Table 4).

**Table 4:** Correlation matrix of BMI and sociodemographic factors Correlation is significant at the 0.05 level (two-tailed).

Variable		BMI	Age in years	Gender	Religion	Class in study	Ethnicity	Education status of the father	Education status of the mother	Occupational status of the father	Occupational status of the mother	Socioeconomic status of the family
BMI	Pearson’s r	—										
	df	—										
	p-value	—										
Age in years	Pearson’s r	0.096	—									
	df	287	—									
	p-value	0.104	—									
Gender	Pearson’s r	0.001	0.067	—								
	df	287	287	—								
	p-value	0.99	0.253	—								
Religion	Pearson’s r	-0.077	-0.006	0.059	—							
	df	287	287	287	—							
	p-value	0.193	0.919	0.32	—							
Class in study	Pearson’s r	0.071	0.721	0.121	-0.005	—						
	df	287	287	287	287	—						
	p-value	0.227		0.04	0.938	—						
Ethnicity	Pearson’s r	0.132	0.055	-0.031	-0.337	0.044	—					
	df	287	287	287	287	287	—					
	p-value	0.025	0.355	0.596		0.451	—					
Education status of the father	Pearson’s r	-0.05	-0.138	-0.056	0.143	-0.159	0.009	—				
	df	287	287	287	287	287	287	—				
	p-value	0.395	0.019	0.347	0.015	0.007	0.883	—				
Education status of the mother	Pearson’s r	0.027	-0.162	-0.008	0.046	-0.187	-0.006	0.59	—			
	df	287	287	287	287	287	287	287	—			
	p-value	0.642	0.006	0.893	0.44	0.001	0.917		—			
Socioeconomic status of the family	Pearson’s r	-0.002	-0.234	0.05	0.161	-0.201	-0.066	0.384	0.372	0.445	0.327	—
	df	287	287	287	287	287	287	287	287	287	287	—
	p-value	0.968	<0.001>	0.397	0.006	<0.001>	0.262	<0.001>	<0.001>	<0.001>	<0.001>	—

Correlation between BMI and factors related to obesity

Correlation analysis was conducted to examine the relationship between BMI and factors related to obesity, including the consumption of fast-food items. The results indicated a significant relationship between BMI and the consumption of processed food items (r = -0.135, p = 0.022) (Table 5).

**Table 5:** Correlation matrix of BMI and factors associated with overweight and obesity Correlation is significant at the 0.05 level (two-tailed).

Variable		BMI	Presence of underlying disease condition	Food preference	Sleeping pattern	Screen time	History of obesity in the family	Playing indoor games	Consumption of processed food	Consumption of beverages
BMI	Pearson’s r	—								
	df	—								
	p-value	—								
Presence of underlying disease condition	Pearson’s r	0.023	—							
	df	287	—							
	p-value	287	—							
Food preference	Pearson’s r	0.009	-0.032	—						
	df	287	287	—						
	p-value	0.879	0.589	—						
Sleeping pattern	Pearson’s r	0.062 287	-0.040 287	-0.103 287	—					
	df	287	287	287	—					
	p-value	0.295	0.503	0.08	—					
Screen time	Pearson’s r	0.066	0.092	-0.017	0.019	—				
	df	287	287	287	287	—				
	p-value	0.26	0.12	0.769	0.75	—				
History of obesity in the family	Pearson’s r	0.089	0.049	-0.05	-0.01	0.036	—			
	df	287	287	287	287	287	—			
	p-value	0.131	0.406	0.394	0.862	0.548	—			
Playing indoor games	Pearson’s r	0.062	0.011	0.018	0.012	0.176	0.089	—		
	df	287	287	287	287	287	287	—		
	p-value	0.290	0.854	0.763	0.835	0.003	0.132	—		
Consumption of processed food	Pearson’s r	0.135	-0.037	0.138	-0.162	-0.168	-0.053	-0.135	—	
	df	287	287	287	287	287	287	287	—	
	p-value	0.022	0.534	0.019	0.006	0.004	0.370	0.022	—	
Consumption of beverages	Pearson’s r	0.102	-0.001	0.105	-0.165	-0.098	-0.076	0.013	0.466	—
	df	287	287	287	287	287	287	287	287	—
	p-value	0.085	0.991	0.073	0.005	0.095	0.200	0.830	<0.001	—

Regression analysis

Variables show a strong relationship with BMI, such as eating processed foods and ethnicity, which were further studied using linear regression and have found a strong link with BMI (processed foods: R = 0.135, R² = 0.0181, F = 5.30, p = 0.022; ethnicity: R = 0.132, R² = 0.0175, F = 5.10, p = 0.025) (Table 6).

**Table 6:** Regression analysis of BMI and processed food, and BMI and ethnicity

Variable	Model	R	R²	Adjusted R²	F	df1	df2	p-value
BMI and processed food	1	0.135	0.0181	0.0147	5.3	1	287	0.022
BMI and ethnicity	1	0.132	0.0175	0.014	5.1	1	287	0.025

BMI and processed food

ANOVA shows a strong link between BMI and processed food (mean square = 63.3, F = 5.30, p = 0.022) (Table 7), whereas the model coefficient shows a t-value of 27.46 (Table 8).

**Table 7:** ANOVA for BMI and consumption of processed food Significance at the 0.05 level

Source	Sum of squares	df	Mean square	Significance F	p-value
Consumption of processed food	63.3	1	63.3	5.30	0.022
Residuals	3,432.7	287	12		

**Table 8:** Coefficients for BMI and processed food Significance at the 0.05 level

Predictor	Coefficient	Standard error	Lower 95%	Upper 95%	t-value	p-value
Intercept	19.583	0.713	18.18	20.9861	27.46	
Consumption of processed food	-0.584	0.254	-1.08	-0.0845	-2.3	0.022

BMI and ethnicity

ANOVA also shows a link between BMI and ethnicity (mean square = 61.1, F = 5.10, p = 0.025) (Table 9), whereas the model coefficient shows a t-value of 9.57 (Table 10).

**Table 9:** ANOVA for BMI and ethnicity Significance at the 0.05 level

Source	Sum of squares	df	Mean square	F-value	p-value
Ethnicity	61.1	1	61.1	5.10	0.025
Residuals	3,435	287	12		

**Table 10:** Coefficients for BMI and ethnicity Significance at the 0.05 level

Predictor	Coefficient	Standard error	Lower 95%	Upper 95%	t-value	p-value
Intercept	14.6	1.525	11.596	17.6	9.57	
Ethnicity	1.77	0.784	0.228	3.31	2.26	0.025

## Discussion

This cross-sectional study intended to determine the prevalence of obesity among schoolchildren in Jharkhand, revealing that 2.8% of the total schoolers were overweight, while no instances of obesity were identified. This data does not align with a study that shows there is a significant prevalence of obesity and overweight among schoolchildren in Jharkhand [8].

Our study result was supported by the 2019 World Obesity Federation report, which estimated that by 2025, 206 million children and adolescents aged 5-19 would be living with obesity, with projections indicating an increase to 254 million by 2030 [13]. Research indicates that more than one million children are likely experiencing obesity across 42 countries, with China at the forefront, followed by India, the USA, Indonesia, and Brazil. Remarkably, only seven of the top 42 countries qualify as high-income nations [14].

In India, obesity among children is increasing gradually. A recent meta-analysis shows obesity among children is 8.4%, whereas overweight was 12.4% among children [15]. The National Family Health Survey 2019-21 (NFHS-5), by the government of India, found that 3.4% of children under five are overweight compared with 2.1% in 2015-2016 [16].

It is noteworthy that the Indian states exhibiting the highest prevalence of child undernutrition are progressively emerging as hotspots for obesity [17]. That is true for Jharkhand State in India.

The NFHS-5 indicates that in Jharkhand, the prevalence of stunting, underweight, and wasting among children under five years old is 39.5%, 22.4%, and 39.4%, respectively. The proportion of women aged 15-49 with a BMI below 18.5 kg/m² was 26.2% [18]. Interestingly, the same report also shows the Ranchi district in Jharkhand has 12,055 obese children. This information supports the findings of the World Obesity Atlas by UNICEF 2022, which forecasts that India will have over 27 million obese children by 2030, accounting for one in ten children worldwide [18].

The factors contributing to childhood obesity are diverse, encompassing genetic, familial, environmental, cultural, and health-related influences [19]. Our investigation revealed a notable correlation between BMI and ethnicity, specifically distinguishing between tribal and non-tribal groups, as well as between BMI and processed food. However, we were unable to identify any additional associations between BMI and sociodemographic factors or other underlying elements that may contribute to obesity in children. Other studies [20] have also found a big difference in the rates of obesity between tribal and non-tribal populations. There is also a link between eating processed foods and being overweight or obese [21].

As childhood obesity can affect a child’s physical and mental health, it can lead to heart problems, non-insulin-dependent diabetes mellitus, bronchial asthma, high blood pressure, obstructive sleep apnea, gastroesophageal reflux disorders, polycystic ovarian syndrome, orthopedic problems, and other related conditions. It can also cause mental health problems like eating disorders, low self-esteem, and depression. Implementing a variety of preventive and therapeutic interventions is essential to reduce the burden of comorbid health conditions [22].

As childhood obesity becomes more ubiquitous and more common among the urban population, children from wealthy families and children who consume more non-vegetarian and processed foods, parents, teachers, policymakers, and healthcare team members should develop targeted interventions to reduce childhood obesity, taking into account the socioeconomic diversity of India. These interventions should not only consider the child’s age and interests but also simultaneously enhance the child’s confidence.

Limitations

This study has several limitations. Participants were selected conveniently from two schools, which could potentially lead to selection bias. The study has only included participants from private schools, which makes it difficult to compare across different social classes and communities. This study does not assess the various underlying factors of obesity and overweight, which could also be considered a potential limitation.

## Conclusions

This cross-sectional study indicates a gradual rise in overweight prevalence among schoolchildren in countries previously known for malnutrition. Furthermore, our investigation did not reveal any correlation between overweight and obesity and the sociodemographic status or educational background of the parents. Our findings reveal a direct association between ethnicity and the consumption of processed food with obesity among schoolchildren in Jharkhand, India, underscoring the necessity for focused interventions from policymakers.
